# Monthly Digest

**Published:** 1895-01

**Authors:** J. M. Walker


### Monthly Digest.

BY MRS. J. M. WALKER.

### DENTAL EDUCATION.

PROF. L. L. SKELTON, in the opening address before the Chicago College of Dental Surgery,* discusses the question: "What Constitutes a Practical Dental Education?" In refutation of a general misconception on this point, arising from a confusion of the terms *practical* with *manual* or *technical* (*theory*, seeming to imply something to be classed among the adornments of the scholar,) he states, as a general proposition, that a practical dental education is necessarily composed of two parts, *first*, the theory; *second*, the manual in its broadest sense; neither can do without the other.

Quoting the universal complaint of the student, that so much time is spent on subjects, the connection of which, with practical dentistry, he is wholly unable to appreciate, Prof. Skelton proceeds to show that a knowledge of the normal furnishes the only landmark by which to recognize the abnormal; that a knowledge of the physiological is necessary for an appreciation of the pathological. To understand the working of a vital machine there must be a knowledge of its construction; hence the study of anatomy, histology, chemistry, etc. The physician and the

*Dental Review, November 15, 1894.

dentist have common ground in the fundamental branches, in which the dentist must be well grounded while in college, in order to make the intelligent application necessary for the success of the practical dentist.

Dr. Louis J. Stephan, in a paper read before the Wisconsin State Dental Society,* urged the importance of the "Dental Education of the Public." He spoke of the work of the medical profession in their endeavors to enlighten the public in regard to sanitary science, hygiene and prophylaxis, and of the necessity of renewed efforts on the part of the dental profession in disseminating a knowledge of the mouth and teeth and their functions, and awakening a general interest in the prevention and arrest of decay and loss of the teeth.

How to disseminate such knowledge is a problem which may well engage the attention of dental organizations.

To forward this object, Dr. Stephan suggests that the relations of the oral cavity to the alimentary canal should be taught in the public schools; that chapters on the teeth and their conditions, and the methods for their protection, should be inserted in the current school text-books on physiology; that at the annual meetings of the State Dental Societies, lectures on these subjects should be given to the public, that Educational Committees should be appointed to confer with School Boards with regard to the attainment of these objects, and last, but not least, that members of the profession should contribute towards defraying the cost of carrying out these measures.

In the President's Address before the Minnesota State Dental Association,† Dr. C. H. Stearns discussed the work of State Societies as a factor in elevating the standard of "Dental Education," suggesting the plan of some special work, to which the attention of the profession throughout the State should be devoted during the ensuing year, the idea being to have issued a syllabus, list of books, points to be looked up, material to be used, etc., as a guide to those wishing to improve themselves in that particular branch; at the next annual meeting the selected

* Dental Review, November 15, 1894.

† Dental Review, November 15, 1894.

subject could be intelligently discussed, and all be prepared to take an active part in the work of the Society. This would constitute a sort of Chautauqua course, taking the place of a Post Graduate course for the many to whom the latter is an impossibility. In the discussion of the address this suggestion was most favorably received, and a committee was appointed to work up the plan. As the result the subject of Pyorrhœa Alveolaris was chosen, and a sum, (not to exceed twenty-five dollars,) allotted to defray the expense of preparing and sending out the syllabus, etc.

Dr. E. C. Kirk, in a paper read before the American Academy of Dental Science (Boston,)* entitled "The Laboratory Method in Dental Education," endorses the increasing adoption of laboratory and individual teaching, in lieu of the didactic and class methods.

By the latter method, he says, the scholar acquires his knowledge second-hand, from mental impressions conveyed orally by the teacher. By the laboratory method he receives his knowledge by direct sense impressions derived from material objects or phenomena.

This has, heretofore, been accomplished in a measure, through the mechanical and operative clinics, but as carried out was inherently bad and little short of malpractice, as the green student was forced to acquire his training upon patients of the dental clinic, and subsequently upon the unfortunates who consulted him during the first years of his practice. These objectionable methods are now being supplanted by "Technic Courses," based wholly upon the laboratory method, in both mechanical and operative dentistry. The fundamental principles upon which this system or method is founded are self-evident truths, and when clearly understood criticism will be disarmed, and the method recognized as holding the highest possibilities for good in its application to dental education.

The discussion of this paper ran largely upon the value of manual training. Dr. Kirk, in closing the discussion, said that he did not emphasize the "laboratory method" solely as a means

* International Dental Journal, December, 1894.

of acquiring manual dexterity and skill, but rather as a means of enabling the student to acquire, at first-hand and for himself, a knowledge of facts from which, to reason scientifically, and therefore correctly; a method of instruction based upon a practical knowledge of objects and phenomena, presented to the mind of the student through the medium of his own perceptive faculties.

DR. H. A. PALMER, in a paper on the "First Permanent Molar,"* emphasizes its importance, the general ignorance of the public concerning it, its usually poor structure and the causes of this inferiority, the disastrous results following its loss, and urges the duty of the profession in "Educating the Public" on these points, that the men and women of the future may rise up and call us blessed.

STOMATOLOGY.

The propriety of the adoption of the title "Academy of Stomatology" by a Society of Dental Practitioners is discussed editorially in the Dental Cosmos.†

The necessity of a more comprehensive term, in view of the immensely broadened field of operations opened to the intelligent dentist in consequence of his increasing knowledge of the systemic relationships of the teeth; the etymological fitness of the word to express all that is now included in the present knowledge of the mouth, its functions, its care in health and its treatment when diseased; the uniformity of the term with the designation of analogous departments of medical practice *e. g.* laryngology, ophthalmology, gynæcology, rhinology, otology, are the principal arguments advanced in favor of the acceptance of the term "Stomatology" to describe the conditions now existing, its adoption being regarded by the editor as a mark of distinct professional advancement.

At the October meeting of the Wistar Biological Association, University of Pennsylvania,‡ PROF. W. D. MILLER delivered a lecture on the "Bacterio Pathology of the Human Mouth and its Significance in Relation to Systemic Diseases," illustrated by a

* Dental Review, November 15, 1894.
† Dental Cosmos, December, 1894.
‡ Dental Cosmos, December, 1894, series of photo-micrographs, showing in detail the morphology of the several groups of pathogenic bacteria which inhabit the human mouth. In his introductory remarks, Prof. Miller dwelt upon the toxic character of the human saliva, due to the presence of the micrococci of the sputum of septicæmia.

The most common results of bacteritic action, in the mouth, are caries of the teeth, pulpitis, alveolar abscesses with, not infrequently, secondary complications of most serious nature—as periostitis, osteomyelitis, caries and necrosis of the bones—many cases terminating fatally through the intervention of septicæmia, meningitis, pneumonia, etc.

From abscesses of the teeth pathogenic bacteria may also find their way into the circulation, proliferating anew at perhaps some remote point of diminished resistance. The pernicious results of swallowing pus and bacteria from abscessed roots and suppurating gums—as in pyorrhœa alveolaris; the causal relation of diseased teeth to glandular swellings; infection from pathogenic germs harbored in the tonsils; the relation of mouth germs to diseases of the middle ear, the germ origin of croupous pneumonia, meningitis, tuberculosis, diphtheria—all show the influence the condition the mouth exerts in determining the general state of health and, in the opinion of Prof. Miller, justify the statement that stomatology is entitled to stand on an equal level with otology, ophthalmology, and the other specialties of medicine.

### SYSTEMIC TREATMENT.

Dr. L. A. Faught read a paper before the New Jersey State Dental Society* urging the duty of the dental practitioner to practice "Systemic Dental Medicine."

The dental organs being a portion of a complicated organism, the very condition which primarily requires manipulative treatment is produced largely by the influences of the general system, and to repair the local organs without treating the system is to invite recurrence of the disease; it is simply effacing effects while ignoring the cause, and a failure to discharge our whole duty. Dr. Faught then classifies dental diseases according to

* Dental Cosmos, December, 1894.

their possibilities of systemic treatment, and gives a short list of medicaments most available, bearing in mind that medicines act on the body at large by one of these methods—either by control of tissue waste and repair; by their direct effects on the various organs; or by their effect on the secretions and excretions. He asks an earnest consideration of the subject, and the prompt and general introduction into dental practice of systemic dental medicine.

### EROSION.

Dr. A. P. Brubaker, in a paper read before the Odontological Society of Pennsylvania,* on the "Causation of Dental Erosion," describes the abnormal appearance of the labial glands, in cases of erosion, gives a brief resume of the anatomy and physiology of the structures involved, and a summary of the normal process of secretion. In cases of erosion, the change from physiological to pathological, in the labial glands, is evidenced by persistent hyperæmia, and a secretion altered in viscidity, and changed from alkaline to an acid reaction. He then investigates the nature of the acid present, its origin, and its capacity for decalcifying the enamel. He dissents completely from the uric acid theory for four reasons:

1st. The excretion of uric acid is not a function of the mucous glands.

2d. The insufficient fluid secretion of the labial glands for the solution of uric acid.

3d. That uric acid is amphoteric in character, and not always an acid product.

4th. That both from its small amount and its feeble combining power, it is incapable of decalcifying enamel.

He then discusses the normal constituents of the secretions of these glands and their possible chemical combinations under abnormal conditions, reaching the conclusion that the acid in question is probably the sodic phosphate, and shows both theoretically and by actual tests, the possibility of the decalcification of enamel in the presence of this acid through the gradual disintegration of the molecules of the calcium phosphate of the tooth. As a method of treatment he suggests the destruction of the diseased glands by electrolysis. At the conclusion of the reading

* International Dental Journal, December, 1894.

Dr. Brubaker passed around for inspection, teeth which had been immersed in a weak solution of the acid sodium phosphate, and which showed small eroded spots and a number of transverse fissures produced by daily friction with the tooth brush.

In the discussion of this paper Dr. C. N. Pierce stated that he had examined the teeth previous to this treatment, and that they had been entirely free from any abrasion.

Dr. Faught thought that the investigations of Dr. Brubaker touched upon the process at one end of the line only, and that uric acid might still be the systemic irritant producing the pathological conditions.

Dr Gaskill thought the same results to the teeth might follow the use of acids in systemic treatment, and reported several cases of marked effects upon the teeth from the use of "microbe killer."

### ANÆSTHESIA.

Dr. E. C. Kirk, at the Academy of Stomatology, at Philadelphia,* read a paper on and demonstrated Dr. Hewitt's method of using "Nitrous Oxide and Oxygen" as an anæsthetic mixture, by means of which true anæsthesia is induced and maintained without asphyxial symptoms. Dr. Hewitt's apparatus consists of a double bag, a mixing chamber and three steel cylinders, of uniform size and appearance, one of which contains oxygen; the other two, which are yoked together with a tube coupling, contain compressed nitrous oxide, considerably more than twice as much nitrous oxide as oxygen being used during the inhalation. The relative proportions of the gases are under perfect control of the operator by means of a dial and movable indicator on the mixing chamber, the valve admitting a constant stream of nitrous oxide and as many proportionate parts of oxygen, through numbered perforations, as the case may require. The valve of the apparatus is operated by a foot-key, leaving both hands free.

Following the reading of the paper a demonstration of Dr. Hewitt's method was giver, the patients being anæsthetized with none of the usual symptoms of asphyxia but all the appearance of normal deep sleep.

---

Cosmos, December, 1894.

